# Low-Fat Diet Designed for Weight Loss But Not Weight Maintenance Improves Nitric Oxide-Dependent Arteriolar Vasodilation in Obese Adults

**DOI:** 10.3390/nu11061339

**Published:** 2019-06-14

**Authors:** Abeer M. Mahmoud, Chueh-Lung Hwang, Mary R. Szczurek, Jing-Tan Bian, Christine Ranieri, David D. Gutterman, Shane A. Phillips

**Affiliations:** 1 Department of Physical Therapy, University of Illinois at Chicago, Chicago, IL 60612, USA; amahmo4@uic.edu (A.M.M.); clhwang@uic.edu (C.-L.H.); mszczurek@uic.edu (M.R.S.); jtbian@uic.edu (J.-T.B.); craneri@uic.edu (C.R.); 2 Integrative Physiology Laboratory, College of Applied Health Sciences, University of Illinois at Chicago, Chicago, IL 60612, USA; 3 Cardiovascular Center, Department of Medicine, Medical College of Wisconsin, Milwaukee, WI 53226, USA; dgutt@mcw.edu; 4 Division of Endocrinology, Diabetes, and Metabolism, Department of Medicine, University of Illinois at Chicago, Chicago, IL 60612, USA

**Keywords:** low-fat diet, weight loss, hypocaloric, isocaloric, obesity, microvasculature, nitric oxide, cardiovascular, flow-induced dilation, acetylcholine

## Abstract

Obesity is associated with microvascular dysfunction. While low-fat diet improves cardiovascular risk, its contributions on microvascular function, independent of weight loss, is unknown. We tested the hypothesis that nitric oxide (NO)-dependent vasodilation in microvessels is improved by low-fat diets designed for weight loss (LFWL) compared to low-fat weight maintenance (LFWM) diet. Obese adults were randomly assigned to either a LFWL diet (*n =* 11) or LFWM diet (*n =* 10) for six weeks. Microvessels were obtained from gluteal subcutaneous fat biopsies before and after the intervention for vascular reactivity measurements to acetylcholine (Ach) and flow, with and without L-NAME or indomethacin. Vascular and serum NO and C-reactive protein (CRP) were also measured. LFWL diet increased flow-induced (FID) and ACh-induced dilation (AChID); an effect that was inhibited by L-NAME. Conversely, LFWM diet did not affect FID or AChID. Indomethacin improved FID and AChID in the baseline and this effect was minimized in response to both diets. Serum NO or CRP did not change in response to either diet. In conclusion, LFWL diet improves microvascular reactivity compared to LFWM diet and increased vascular NO contribution to the improved microvascular dilation. These data suggest that weight reduction on low fat diet is critical for microvascular health.

## 1. Introduction

Obesity is a common and serious disease that affects more than one-third (36.5%) of the U.S. adults and over 650 million adults worldwide [1]. The most common preventable leading causes of death in obese adults are related to cardiovascular disease (CVD) [2]. The risk of developing CVD is increased by up to 10-fold in individuals with obesity compared to the general age-matched population [3]. It has been estimated that 45% of American women and 30% of American men follow diets to lose weight [4]. However, dietary advice has been conflicting and definitive data to justify the contribution of various diet strategies for cardiovascular health are lacking and warranting further investigations.

The American Heart Association (AHA) has recommended a low-fat diet for cardiovascular health promotion [5]. While in most of the studies that implemented low-fat diet, improvements in some cardiovascular risk factors such as lipid metabolism and oxidative stress were reported, findings that are related to actual vascular measurements have been lacking or conflicting. For example, while Phillips et al. [6] have shown that low-fat diet improved arterial flow-mediated dilation (FMD), contradictory results have been reported by Volek et al. [7], who reported FMD decreases with a low-fat diet and Keogh et al. [8] who demonstrated no effect of low-fat diet on vascular function. Considering these conflicting reports, it is easy to conclude that some additional factors must play a key role in vascular outcomes of the low-fat diet and may reveal contributing mechanisms.

Growing evidence suggests that microvascular dysfunction is a powerful predictor of CVD risk in obesity likely through elevated peripheral resistance and attenuated flow [9,10,11]. Framingham investigators have demonstrated that aortic stiffness may be mediated in part by pathways that include microvascular damage and remodeling which emphasizes the role of microvasculature in the development of CVD [12]. Our previous studies have shown that microvascular function is compromised in obese individuals and could be improved by lifestyle interventions such as weight loss interventions [13,14]. In contrast to the contradictory findings from low-fat diet studies, weight loss interventions consistently reported vascular improvements and enhancements of cardiometabolic risk factors [15,16,17,18]. On the other hand, the available evidence regarding the effect of weight maintenance on cardiovascular risk has been collected by weight loss interventions that were followed by weight maintenance. Accordingly, the mere effect of weight maintenance on cardiovascular risk is not completely understood.

Previous studies reported direct correlations between the magnitude of body weight loss and the reduction in cardiovascular risk. Dietary-induced weight loss has been shown to reduce cardiovascular risk by ~ 20% while greater weight loss after bariatric surgery reduced the risk by over 30% [19]. Even within dietary-induced weight loss interventions, the effect on reducing cardiovascular risk varies greatly among different diets such as energy restriction diet, low fat diet, low-carbohydrate diet, high protein diet, or low glycemic index diet. This variation might indicate an interaction between the actual body mass reduction and the type of diet that has been followed. Therefore, it is not clear whether the vascular outcome of a low-fat diet is confounded by the cardiovascular benefits of caloric restriction-mediated weight loss.

Short term LF diet interventions induce minimal, if any, weight loss, however, they effectively enhance lipid profiles and insulin sensitivity, both are possible intermediate mechanisms for vascular improvements [20,21,22,23]. While reducing low density lipoproteins (LDLs) salvages endothelial function by reducing inflammation and reactive oxygen species (ROS) production, increasing insulin sensitivity enhances flow-induced NO production and subsequently improves vasodilation [13,24,25]. However, the extent to which these mechanisms contribute to vascular improvements in response to LF diet with no caloric restriction (WM) versus low-fat diet accompanied by caloric restriction (WL) is not completely understood. Accordingly, the purpose of the current study was to test the hypothesis that the effect of LFWL diet and LFWM diet on microvascular endothelial-mediated dilation might differ despite possible similar beneficial effects on lipid profiles and insulin sensitivity. This proposed difference might be attributed to the synergistic effect of LF and restricted caloric aspects of the LFWL diet compared to LFWM diet that has no caloric deficit. To this end, FID and AchID measurements were performed in adipose tissue-isolated arterioles from two groups of obese subjects who consumed either LFWL or LFWM diets for six weeks. The mechanistic contribution of NO and prostaglandins to arteriolar vasoreactivity at baseline and the end of the intervention were also assessed. Systemic biomarkers of inflammation (CRP) and NO bioavailability were assessed in the blood of all participants as well as lipid values and measurements of insulin sensitivity. Finally, anthropometric measurements including body weight, body mass index (BMI), and DEXA scanning-assessed body fat percentage were performed.

## 2. Subjects and Methods

### 2.1. Human Participants

**Figure 1:**
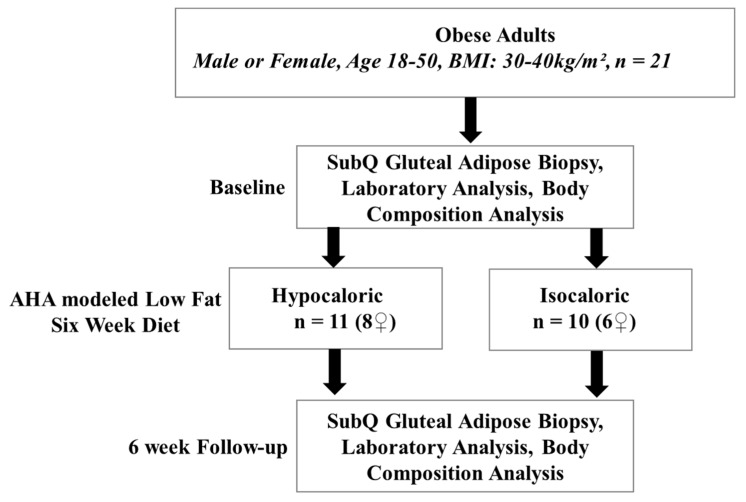
Study flow chart.

A cohort of 21 participants (14 females and seven males) was recruited from the Greater Chicago area. The study flowchart detailing subject randomization, dietary intervention, and follow up is displayed in Figure 1. To be included in the study participants had to be 18 to 50 years old with a body mass index (BMI) that ranges between 30 and 39.9 kg/m^2^. Smokers, diabetic, or CVD patients were excluded. Participants were excluded if they had a history of eating disorders, followed a diet, gained or lost >6 kg in the past year; had a history of chronic illness, cardiac, pulmonary, liver, or kidney abnormalities, or hypertension. Also, pregnant, lactating, peri- and post-menopausal women were excluded. All human procedures in this study were approved by the Institutional Review Board of the University of Illinois at Chicago. Written informed consents were obtained from all participants before participation.

### 2.2. Study Design

A 6-week low-fat dietary intervention was applied to test the study objectives. The dietary intervention was modeled after the AHA low-fat diet (50% carbohydrate, 30% protein, 20% fat) [5]. All meals were prepared by the study staff. Subjects were randomly assigned to either a hypocaloric diet designed for weight loss or an isocaloric diet designed for weight maintenance designed at resting energy needs. Caloric needs were assessed via the Mifflin equation [26] and the WL diet was designed to induce 500 calorie/day deficit based on each participant’s resting energy needs. Both WL and WM diets followed the AHA low-fat dietary composition mentioned above. LFWL diet was consumed by participants for four weeks, followed by LFWM for the final two weeks in order to avoid the confounding effects of active weight loss on FID and other inflammatory markers [6]. All meals were prepared in the metabolic kitchen of the UIC College of Applied Health Sciences and distributed to participants one or twice a week. The participants were instructed to consume only the meals that were prepared and provided by the study team and to refrain from consuming alcoholic beverages throughout the study. Compliance with the dietary instructions was assessed via daily recording items that were added or missed from the dietary plan. Subjects were also instructed to maintain their usual level of physical activity throughout the study. At the baseline (week 1) and the end of the intervention (week 6), we measured subject body composition (using dual-energy X-ray absorptiometry (DEXA)), height, weight, BMI, waist circumference, blood pressure, cholesterol, lipid profile, glucose, and insulin. Also, at weeks 1 and 6, plasma samples were collected to measure levels of adipocytokines, and subcutaneous gluteal adipose tissue biopsies were performed to measure adipose microvascular reactivity to Ach (10^−9^–10^−4^ mol/L) and Flow (Δ10–Δ100 cmH_2_O). Before clinical procedures and measurements, subjects were instructed to fast overnight for 10–12 h and refrain from exercise training beyond their regular activities. All participants recorded their diet and exercise behaviors for the three days prior to the testing day and were asked to refrain from consuming caffeine-containing beverages (12 h prior). For all participants, a pedometer was worn with the step count recorded daily on an exercise log.

### 2.3. Subcutaneous Adipose Tissue Biopsy

A gluteal biopsy procedure was performed for all participants as we previously described [27]. Briefly, the skin over the gluteal area was locally anesthetized using lidocaine (2%), and an incision (0.5–1 cm) was made under sterile conditions to expose subcutaneous fat which extrudes through the incision site. The sample was placed in cold HEPES (4-(2-hydroxyethyl)-1-piperazineethanesulfonic acid) buffer with a pH of 7.4 for FID experiments that were conducted the same day of biopsy collection [13].

### 2.4. Microvascular Preparation

After dissection of excess fat and connective tissues, isolated adipose tissue resistance arterioles were prepared for measuring changes in the internal diameter in response to flow, or Ach as previously described [13,28,29]. Arterioles were cannulated in an organ perfusion chamber using glass micropipettes (outer tip diameter ≤40 μm) that are pre-filled with cold Krebs buffer (physiological salt solution) that contains the following ingredients (mM) 123 NaCl, 4.4 KCL, 2.5 CaCl_2_, 1.2 MgSO_4_, 20 NaHCO_3_, 1.2 KH_2_PO_4_, and 11 glucose. Arterioles were secured from both ends using 10-0 nylon Ethilon monofilament suture. Cannulated arterioles were then transferred to the stage of an inverted microscope with an attached video camera that is, in turn, connected to a video monitor and a video measuring device model VIA-100 (Boeckeler, Madison, WI, USA). The organ chamber was continuously perfused with heated Krebs buffer solution (pH = 7.4 ± 0.05, Po_2_ = 140 ± 10 mmHg, 37 °C) and supplied with a mixture of 21% O_2_, 5% CO_2_, and 74% N_2_. Each end of the glass micropipettes, to which vessels were secured, was connected via 0.050 inches (ID) silicon tubes to a Krebs buffer-containing reservoir. Intraluminal pressure gradient was created by changing the distance between the reservoirs in equal and opposite directions [30]. Intraluminal pressure of 60 cm H_2_O was maintained inside the arterioles for 30 min.

### 2.5. Flow-Induced Dilation Measurements

Arterioles were pre-constricted with endothelin-1 (Peninsula, San Carlos, CA, USA) and those that did not constrict beyond 30% were excluded from the study. The steady-state internal diameter was measured before and during intraluminal flow corresponding to pressure gradients of 10–100 cmH_2_O or log increases in the concentration of acetylcholine (ACh; 10^−9^–10^−4^ M) [28]. This approach was repeated after incubating vessels with the NOS (nitric oxide synthase) inhibitor (L-NAME; 10^−4^ M), or the cyclooxygenase enzyme inhibitor, indomethacin (10^−5^ M) for 30 min. When possible, one arteriole from each biopsy was used for the whole protocol (baseline, LNAME, and indomethacin) measurements. However, if an arteriole did not constrict or dilate properly in response to endothelin-1 and papaverine, respectively, another arteriole from the same adipose tissue biopsy was used. At the end of each experiment, papaverine (10^−4^ M) was used to detect maximal vasodilation. The reported percentage of vasodilation was calculated as the % increase in diameters after each treatment condition relative to the pre-constricted state.

### 2.6. Nitric Oxide Measurements in Isolated Arterioles

Nitric oxide generated by the isolated microvessels was measured as previously described [14] using Enzo Life Sciences NO Detection Kit (ThermoFisher Scientific, Waltham, MA, USA). These measurements were performed in vessels that were cannulated in the organ chambers and prepared as we described above in the microvascular preparation section. Vessels were incubated with the NO detection reagents while being exposed to a pressure gradient of Δ60 cm H_2_O in order to stimulate NO production. This procedure was followed by excising the vessels, washing and mounting them on microscopic coverslips, and imaging the vessels immediately using fluorescence microscopy (Eclipse TE 2000, Nikon, Japan) at 650/670 nm; the recommended wavelength for NO detection. NO detection was performed on freshly isolated vessels. In order to maintain consistency among different measurements, staining protocol, incubation period in NO detection reagents, microscopic settings, excitation and emission wave lengths and time of exposure were fixed in all experiments. We then analyzed the acquired images for the intensity of the fluorescent signal in arbitrary units using NIH Image J software (NIH, Bethesda, MD, USA).

### 2.7. Serum Nitric Oxide and C-Reactive Protein (CRP) Measurements

Nitrates and nitrites are stable end-products of NO metabolism that we measured in serum using the Griess reaction (Cayman Chemicals, Ann Arbor, MI, USA) as indicators of NO levels [31]. Measurements were performed following the manufacturer protocol. First, the nitrate fraction in the serum was transformed to nitrite via the enzyme nitrate reductase. This was followed by adding the Griess reagents that subsequently transformed nitrites into dark-colored, easily-detectable azo compounds. Color absorbance was determined at 540 using a multimode plate reader (Molecular Devices, San Jose, CA, USA). C-reactive protein (CRP) concentrations were quantified using high sensitivity ELISA kit (Crystal Chem, Elk Grove Village, IL, USA). Following the manufacturer’s instructions, 20 µL of the pre-diluted serum samples (1:20), blank, and standards were added to the antibody-coated 96-well plate and incubated at room temperature on a shaker at 200 rpm for 30 min. Wells were then aspirated, washed, and 100 µL of the working HRP solution were added to each well and incubated at room temperature for 15 min. Wells were aspirated and washed again and 100 µL substrate solution was added to each well and incubated for 15 min at room temperature after which the reaction was stopped by the stop solution, and the optical density was measured using a SpectraMax M Series multimode plate reader (Molecular Devices, San Jose, CA, USA) at 450 nm.

### 2.8. Cardio-Metabolic Risk Factor Assessment

Serum levels of total cholesterol, triglycerides, low density lipoproteins (LDL), high density lipoproteins (HDL) were measured via enzymatic kits (Roche Diagnostics, Indianapolis, IN, USA). Systolic and diastolic blood pressure measurements were obtained in participants after a 5-min rest according to AHA guidelines [32]. Fasting glucose and insulin concentrations were measured via the glucose oxidase enzymatic assay (Beckman II autoanalyzer) and radioimmunoassay (Linco Research, St. Charles, MO, USA), respectively.

### 2.9. Statistical Analysis

All measures are presented as mean ± standard error. Paired *t*-test was used to compare variables at the pre and post intervention states in each dietary group. The differential effect of the dietary intervention was assessed by using analysis of covariance (ANCOVA) to control for baseline differences between the two groups (LFWL and LFWM). Pearson correlation analysis was used to determine relationships between variables. Statistical significance was set at *p* < 0.05. Data were analyzed using SPSS software (version 18.0; SPSS Inc, Chicago, IL, USA). Post hoc power analysis was performed to find the observed power using post-diet changes in the arteriolar vasodilation as the primary outcome (post hoc power = 0.55).

## 3. Results

### 3.1. Effect of LFWL and LFWM Diet on Body Composition and Cardiometabolic Risk Factors

Table 1 represents the baseline characteristics of the study participants including anthropometric and metabolic variables. Body weight, BMI, and diastolic blood pressure decreased significantly in response to the LFWL diet; however, no significant changes were found in the LFWM group. Interestingly, lipid profile changed significantly in both groups; total cholesterol decreased by an average of 6% in both groups and LDL decreased by 4% in the LFWL group and 10% in the LFWM group. Both groups experienced significant reductions (16–17%) in the HDL levels. Fasting morning insulin was reduced by 47% in the LFWL group and 25% (marginally significant) in the LFWM; however, HOMA-IR (homeostatic model assessment for insulin resistance) reduction was significant in both groups. No significant changes were noticed in the body fat percentage, systolic blood pressure, triglycerides, or glucose in either group. Daily average step count with pedometer was not significantly different between the two groups throughout the intervention period (LFWL = 6680; LFWM = 6826; *p* = 0.6).

### 3.2. Effect of LFWL and LFWM Diet on Flow and Ach-Induced Dilation

**Figure 2:**
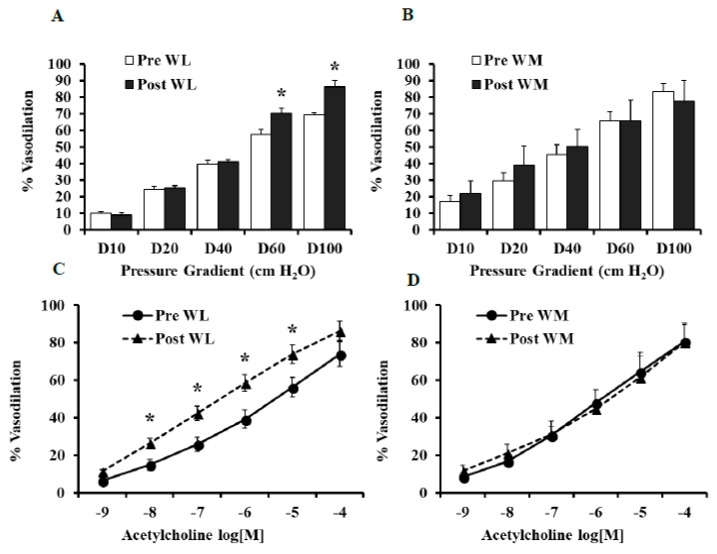
Percent vasodilation in isolated adipose tissue arterioles at 0 (pre) and 6 weeks (post) from WL or WM diet. FID measurements corresponding to increasing intraluminal pressure gradients of 10–100 cmH_2_O (**A** and **B**). AchID measurements corresponding to increasing concentrations of Ach (10^−9^ to 10^−4^ M) (**C** and **D**). All measurements are presented as means ± SE. * (*p* < 0.05) for comparing the pre- vs. post-intervention states.

The pre-intervention endothelial-mediated vasodilation of isolated adipose tissue arterioles was similar in the LFWL and LFWM groups as determined by FID and Ach. Isolated adipose tissue arterioles demonstrated improved FID after the LFWL intervention; % of maximum dilation (MD) at ∆60 cmH_2_O, that mirrors the mean of physiological pressure in vivo, was increased by 21.5% (*p* = 0.03) relative to pre-intervention states (Figure 2A). Isolated adipose tissue arterioles from participants in the LFWM diet did not show any significant differences in the FID at the end of the trial compared to baseline measurements (Figure 2B). These findings were reproduced by exposing isolated arterioles to increased concentrations of ACh (Figure 2C,D), supporting the improved endothelial-dependent vascular reactivity in response to the LFWL diet and the absence of any improvements in response to the LFWM diet. We observed negative correlations between FID and BMI (*r* = 0.4, *p* = 0.01) and total cholesterol (*r* = 0.3, *p* = 0.02). Table 2 represents the FID and AchID measurements in both the LFWL and LFWM groups at the pre- and post-interventions states along with statistical analyses of the magnitude of change in response to each diet after controlling for baseline differences (ANCOVA).

### 3.3. Effect of LNAME and Indomethacin on Flow and Ach-Induced Dilation in LFWL Group

**Figure 3:**
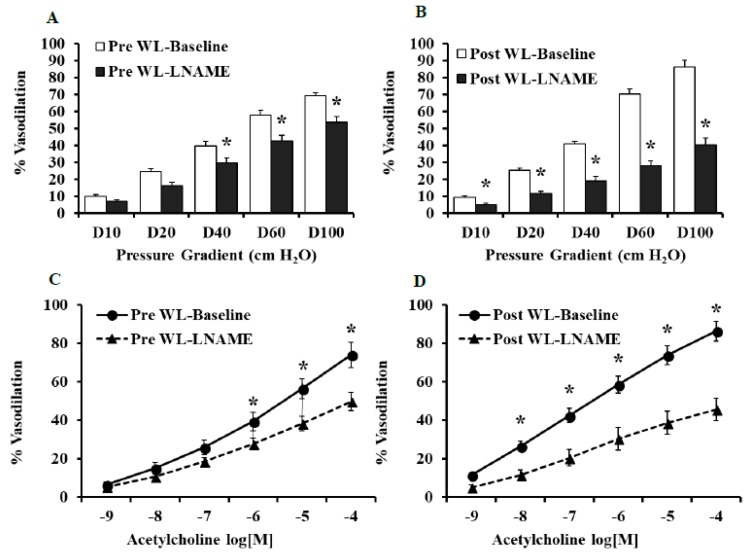
Effects of LNAME on arteriolar FID and AchID at the pre- and post-WL-intervention states. FID measurements corresponding to increasing intraluminal pressure gradients of 10–100 cmH_2_O in the presence or absence of LNAME (10^−4^ M) (**A** and **B**). AchID measurements corresponding to increasing concentrations of Ach (10^−9^ to 10^−4^ M) in the presence and absence of LNAME (10^−4^ M) (**C** and **D**). All measurements are presented as means ± SE. * (*p* < 0.05) for comparing baseline and LNAME in (**A**,**B**) and the pre- vs. post-intervention states in (**C**,**D**).

**Figure 4:**
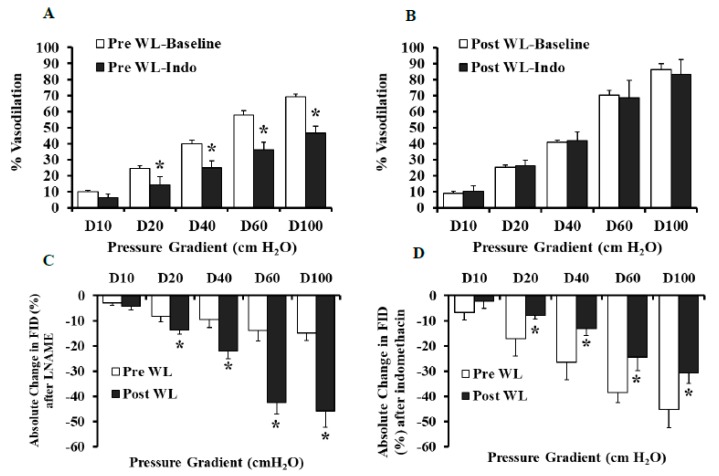
Percent vasodilation and absolute changes in arteriolar FID in response to indomethacin and LNAME at the pre- and post-WL-intervention states. FID measurements corresponding to increasing intraluminal pressure gradients of 10–100 cmH_2_O in the presence and absence of indomethacin (10^−5^ M) (**A** and **B**). Absolute changes in FID were measured in response to LNAME (10^−4^ M) or indomethacin (10^−5^ M) incubation for 30 min (**C** and **D**). All measurements are presented as means ± SE. * (*p* < 0.05) for comparing baseline and indomethacin in (**A**,**B**) and the pre- vs. post-intervention states in (**C**,**D**).

Compared to baseline, FID was reduced in the presence of L-NAME in the pre-intervention and the post-intervention states (Figure 3A,B). However, the L-NAME-induced impairment of FID was more significant in arterioles isolated after the LFWL dietary intervention compared to those isolated before it across all pressure gradients; % of MD at ∆60 cmH_2_O in Pre-LFWL was −14% and in Post-LFWL −43% (*p* < 0.01, Figure 4C), suggesting an increased dependence of FID on NO after the LFWL diet. These findings were recapitulated by exposing isolated arterioles to increased concentrations of ACh (Figure 3C,D). Arteriolar FID following LNAME correlated positively with body weight (*r* = 0.05, *p* = 0.000), BMI (*r* = 0.4, *p* = 0.01), waist circumference (*r* = 0.5, *p* = 0.000), systolic blood pressure (*r* = 0.3, *p* = 0.03), fasting inulin (*r* = 0.4, *p* = 0.003), and HOMA-IR (*r* = 0.4, *p* = 0.002). These correlations may indicate a lack of FID dependence on NO as body weight, blood pressure, and insulin resistance increase. Indomethacin reduced the FID significantly in the adipose tissue arterioles isolated before the intervention. However, no changes were found in the arterioles isolated at the end of the intervention (Figure 4A,B), indicating a reduced dependence of FID on the cyclooxygenase and prostaglandin pathway after the LFWL diet. This is also evident in Figure 4D where the absolute changes in FID after indomethacin compared to baseline measurements were calculated before and after LFWL diet.

### 3.4. Effect of L-NAME and Indomethacin on Flow and Ach-Induced Dilation in LFWM Group

**Figure 5:**
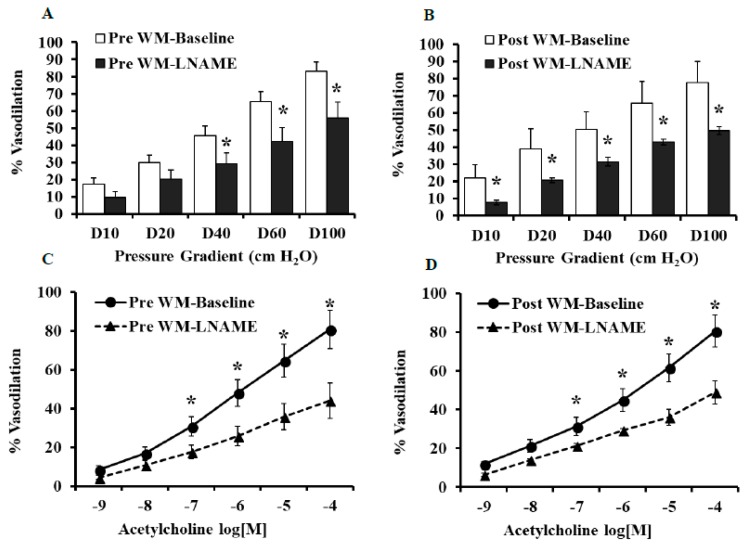
Effects of LNAME on arteriolar FID and AchID at the pre- and post-WM-intervention states. FID measurements corresponding to increasing intraluminal pressure gradients of 10–100 cm H_2_O in the presence or absence of LNAME (10^−4^ M) (**A** and **B**). AchID measurements corresponding to increasing concentrations of Ach (10^−9^ to 10^−4^ M) in the presence and absence of LNAME (10^−4^ M) (**C** and **D**). All measurements are presented as means ± SE. * (*p* < 0.05) for comparing baseline with LNAME.

**Figure 6:**
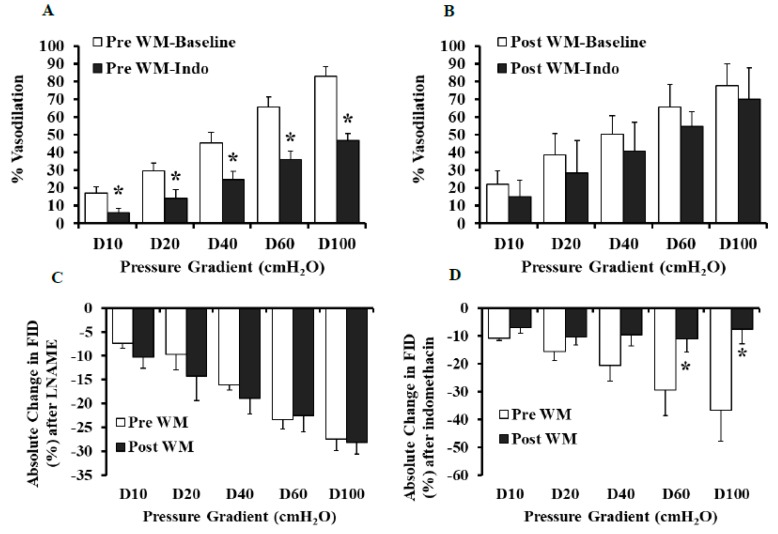
Percent vasodilation and absolute changes in arteriolar FID in response to indomethacin and LNAME at the pre- and post-WM-intervention states. FID measurements corresponding to increasing intraluminal pressure gradients of 10–100 cmH_2_O in the presence and absence of indomethacin (10^−5^ M) (**A** and **B**). Absolute changes in FID were measured in response to LNAME (10^−4^ M) or indomethacin (10^−5^ M) incubation for 30 min (**C** and **D**). All measurements are presented as means ± SE. * (*p* < 0.05) for comparing baseline and indomethacin in (**A**,**B**) and the pre- vs. post-intervention states in (**C**,**D**).

Inhibition of the NO pathway using LNAME resulted in significant reductions in the FID and Ach-ID in the arterioles isolated before and after the LFWM dietary intervention (Figure 5A–D). However, in contrast to what we reported above in the LFWL intervention, there were no differences in the NO dependence between the pre and post LFWM intervention vascular reactivity measurements (Figure 6C). Interestingly, the dependence on the prostaglandin pathway was reduced after the LFWM diet similar to what we reported earlier for the LFWL group. This was evident by the abolishment of indomethacin-mediated reductions in the FID in the post-LFWM compared to the pre-LFWM measurements (Figure 6D). Table 3 represents absolute changes in the FID after LNAME and indomethacin treatment relative to baseline in both the LFWL and LFWM groups at the pre- and post-intervention states along with statistical analyses of the magnitude of change in response to each diet after controlling for baseline differences (ANCOVA).

### 3.5. Flow-Induced NO Fluorescence in LFWL and LFWM Groups

**Figure 7:**
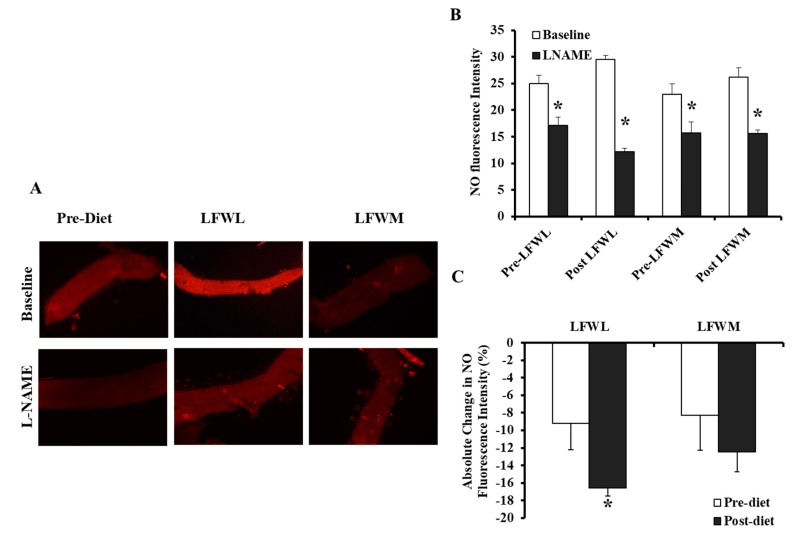
NO production in isolated adipose tissue arterioles. (**A**) Representative fluorescence microscopy images of NO generation conditions and after LNAME incubation (10^−4^ M) for 30 min. (**B**) NO fluorescent signals were measured and expressed in arbitrary units using NIH Image J software. (**C**) Absolute changes in arteriolar NO fluorescence after LNAME (10^−4^ M) incubation in post-WL and post-WM groups relative to pre-diet NO measurements. All measures are represented as means ± SE. * (*p* < 0.05) for comparing baseline with LNAME in (**B**) and the pre- vs. post-intervention states in (**C**).

In addition to measuring the FID, flow-induced NO production was measured in the isolated adipose tissue arterioles using fluorescent NO indicators as described in the methods. The signal intensity was measured using the NIH image J software. Figure 7 shows that NO production increased by 20% after LFWL diet compared to only 6.5% after LFWM. Pre-incubation with LNAME reduced NO production by 34%, 60%, and 40% in the baseline (pre-diet), post-LFWL, and post-LFWM arterioles, respectively.

### 3.6. Effect of LFWL and LFWM Diet on Serum NO and CRP

**Figure 8:**
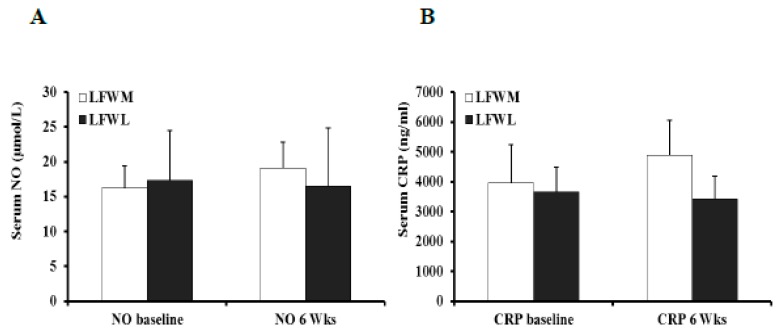
Serum NO (nitrate/nitrite) (**A**) and CRP levels (**B**) at the pre- and post- WL and WM intervention states. All measures represent the means ± SE.

There was no effect of either diet on NO or CRP, predictive markers of vascular function and systemic inflammation, respectively (Figure 8A,B). However, a statistically significant negative correlation was observed between serum NO levels and body fat percentage (*r* = −0.3, *p* = 0.029). Serum levels of CRP correlated positively with body fat percentage (*r* = 0.5, *p* = 0.001), heart rate (*r* = 0.4, *p* = 0.002), fasting glucose (*r* = 0.3, *p* = 0.04), and HOMA-IR (*r* = 0.3, *p* = 0.04).

## 4. Discussion

This study is the first to show that hypocaloric low-fat diet demonstrated benefits over isocaloric low-fat diet on microvascular function in humans. More specifically, we observed an improvement in the FID and Ach dilation of subcutaneous tissue-isolated arterioles in obese adults after six weeks of a hypocaloric low-fat diet that was designed for weight loss. Furthermore, flow-induced NO production in the isolated arterioles and the arteriolar dependence on NO for vasodilation increased significantly in response to the hypocaloric LFWL diet. In contrast, no significant changes in FID, arteriolar NO production, or arteriolar dependence on NO could be observed in response to the isocaloric weight maintenance diet. There was an evident reduction in BMI and diastolic blood pressure after LFWL diet, yet, both diets favorably impacted some cardiovascular risk factors such as total cholesterol, LDL, and insulin levels in the blood.

Obesity is an independent risk factor for cardiovascular diseases such as atherosclerosis and hypertension and metabolic diseases such as insulin resistance and diabetes [33]. Medical, dietary, and surgical weight reduction strategies have been shown to improve endothelial-dependent flow-mediated dilation (FMD) in obese patients [34]. Previous data by our research group and also by others have shown that not all weight reduction diets are similar regarding their effect on vascular function. Phillips et al. [6] have reported greater cardiovascular benefits of low-fat over low-carbohydrate diet in obese individuals. It has also been reported that different low-fat diets might induce different vascular outcome, e.g., a study by Keogh et al. [35], have shown that low-fat diet that is supplemented with high saturated fat (27 g/day), impaired endothelial-mediated vasodilation. Similarly, subjects on low-fat/high-carbohydrate (18% fat; 65% carbohydrate) diet demonstrated elevated cardiovascular risk as evidenced by increases in their triglycerides and total cholesterol levels [35]. Varady et al. [36] demonstrated that weight loss resulting from a low-fat diet improved FMD, while weight loss resulting from a high-fat diet impaired FMD. Furthermore, previous studies showed that endothelial function correlated strongly with fasting blood glucose but not with body weight [37]. Therefore, it is not clear whether caloric restriction or dietary macronutrients contributes more to vascular outcomes. In the current study, we examined the interactive effect of low-fat diet and caloric restriction on microvascular function compared to a low-fat diet that does not induce any weight reduction.

Flow-induced dilation is an essential endothelial mechanism that regulates vascular tone and local blood flow to the tissue. In large arteries, this mechanism is critical for preventing atherosclerosis; however, in microvessels, it plays a vital role in controlling blood pressure through the contributions to vascular resistance [38,39]. FID in subcutaneous arterioles is primarily mediated through the endothelial-mediated release of NO [40]. Other vasoactive mediators such as hydrogen peroxide (H_2_O_2_) and prostacyclin (PGI_2_) might also contribute to FID yet to a lesser extent than NO [41]. Many of the studies that examined the effect of low-fat diets on microvascular function have been performed in animal models. In the current study, we evaluated the effect of a low-fat diet on NO-mediated FID in isolated human arterioles. We observed a comparable degree of LNAME-induced FID reduction between the LFWL and the LFWM groups in the pre-intervention states. However, the most interesting comparison in this study is the LNAME and indomethacin-mediated FID reductions in these two dietary groups, post-intervention, which is reflective of low-fat diet-induced modifications in NO and prostaglandin pathways.

In the present study, we demonstrate that microvascular benefits of having a diet that is low in fat and simultaneously hypocaloric to induce weight loss exceeded those of a low-fat diet that does not induce weight loss, indicating a possible interactive effect of both regimens. These findings are consistent with the FMD data in medium-sized arteries that have been reported by Phillips et al. [6]. However, they are in contrast to what has been shown by Volek et al. [7] who reported a lack of macrovascular FMD improvements in response to a low-fat diet in overweight individuals with moderate dyslipidemia. The reason for these conflicting data remains unclear especially that the average weight loss in the low-fat fed subjects was very comparable in the two studies (~4–5 kg). One possible reason for this discrepancy could be related to the different approach that has been used in these studies; while in Phillips et al., [6] meals were prepared by a dietitian and provided to subjects (similar approach has been used in the current study), in Volek et al. [7], only dietary instructions were provided which might have resulted in unsupervised, uncontrolled macronutrient ratios in the meals consumed by the participants. In general, inconsistencies among studies that investigate the effect of diet on vascular function are mostly attributed to lack of control over the macronutrient proportions consumed by study subjects or the inclusion of other macro- or micronutrients that have been reported to modify vascular outcomes such as dietary fiber [42], nitrite-rich green vegetables, and caffeinated beverages. On the other hand, sources of variations in weight loss studies might be related to changes in body composition and fat distribution. For example, a growing body of evidence proposed a strong relationship between visceral obesity and impaired FMD [43,44,45,46,47].

One of the mechanisms by which low-fat diet-mediated weight loss might improve microvascular function is by improving insulin sensitivity. Insulin resistance and hyperinsulinemia are hallmarks of obesity and contribute to the observed impairments in both macro-and-microvascular function in obese individuals [24]. Insulin is a vasoactive hormone that regulates vascular homeostasis by maintaining a balance between endothelial-derived vasodilators and vasoconstrictors. Insulin resistance and the associated hyperinsulinemia disturb this balance favoring the vasoconstrictive arm [48,49,50]. In the current study, we observed significant reductions in HOMA-IR in response to both LFWL and LFWM diets indicating a role of low-fat diet in improving insulin sensitivity in obese subjects even in the absence of any significant weight loss. This effect is consistent with what has been published by other low-fat dietary interventions in insulin-resistant people [51]. Also, a similar effect on insulin sensitivity has been reported by weight loss studies irrespective of weight loss strategy [52], suggesting a role of the sole reduction of fat mass on improving insulin sensitivity. According to these studies, we might suggest that either low-fat diet or caloric restriction-induced weight loss could stand alone as an insulin sensitizer and that applying both approaches might augment this effect. In support of this assumption, our data showed greater insulin reduction (47%) in LFWL diet compared to LFWM diet (25%).

Improvements in insulin sensitivity are also expected to be accompanied by enhanced production of flow-induced NO in blood vessels. In this study, despite the lack of a systemic increase in serum NO in either dietary arms, there was a measurable improvement in flow-induced arteriolar NO production in the LFWL group. Furthermore, the LNAME-mediated reduction in FID was augmented post LFWL relative to the baseline state, indicating increased FID dependence on NO and improved vascular responses to flow in this dietary group. On the other hand, the LFWM group did not demonstrate any improvements in these measurements which may lead us to suggest that caloric restriction is an integral part of dietary-mediated vascular health promotion. This assumption is supported by several animal studies that suggested mechanisms such as reductions in inflammatory cytokines and oxidative stress and enhancements in leptin production and lipid metabolism as caloric restriction-induced changes that improve NO bioavailability and vascular function [53,54,55]. We and others have shown that blood levels of NO are lower in obese people compared to lean, healthy peers [13,31]. Weight loss either via surgery, exercise training, or caloric restriction resulted in improvements in vascular function and NO bioavailability [56]. A moderate caloric restriction intervention by Korybalska et al. [57] has shown that the magnitude of body weight reduction was the primary predictor for NO changes in a group of 41 obese women. Therefore, the lack of systemic NO changes in our study could be related to the moderate reduction in body weight achieved by our participants and that longer interventions or substantial reductions in body weight are required to observe significant improvements in NO systemic bioavailability. In addition, it is possible that flow-mediated improvements in NO production locally in arterioles are earlier events than systemic NO improvements and that the former could be detected after modest weight reduction.

Other mechanisms that may account for improved endothelial function during weight loss on a low-fat diet include systemic reductions in inflammation. The link between obesity and vascular dysfunction is directly related to the accumulation of dysfunctional adipose tissue that secretes several pro-inflammatory cytokines [58]. Reduction in fat mass may contribute to reduced inflammation and subsequently improved vascular health. Also, low-fat diet apart from weight loss lowered systemic inflammatory biomarkers in patients with obesity, diabetes, and coronary heart disease [59,60,61]. However, in our subjects, levels of the inflammation biomarker CRP were not considered to be high at the baseline nor were they altered by dietary intervention. These findings might rule out systemic inflammation as a possible intermediate factor for the LFWL-mediated microvascular FID improvements in our study. Cyclooxygenase (COX)-derived metabolites contribute to the impaired baseline arteriolar FID in both WM and WL groups. This contribution is diminished following the intervention in both groups. Reasons are not clear but this could indicate a role for local inflammation on microvascular function that is not of an adequate magnitude to be detected systemically. COX-derived metabolites, prostaglandins, and thromboxanes play a critical role in modulating vascular reactivity in both physiological and pathological conditions [62] and previous studies have shown that under certain pathological conditions, endothelial dysfunction might be induced as a result of augmented COX enzyme activity and COX-derived metabolite generation [63,64].

Improvements in lipid profiles are expected to contribute to enhancements of microvascular function. In the current study, total cholesterol and LDL decreased significantly in both dietary interventions. However, FID improvements were achieved in the LFWL group only. These findings might suggest that enhanced lipid profiles contribute to FID improvements conditioned upon an actual weight loss. Despite the favorable changes in lipid profiles that were achieved in both dietary arms, paradoxical reductions in HDL were observed, but the significance of this is unclear. Similar findings have been reported in earlier studies, and this phenomenon has been suggested to result from low-fat diet-mediated reductions in apolipoprotein A-1, the major protein component of HDL [65,66,67]. Even though the lower level of HDL cholesterol is a risk factor for the development of CVD, an association between low-fat diet and increased CVD risk has not been established. In efforts to clarify this phenomenon, some studies demonstrated that despite the overall reduction in HDL in response to low-fat diet, HDL/apolipoprotein subtypes that are responsible for cholesterol metabolism were functionally preserved [68,69]. Another lipid profile component that has an established long-standing association with CVD is the plasma level of triglycerides. Yet, studies that measured triglycerides in response to low-fat diets have shown a lot of discrepancies. For example, a meta-analysis of 19 studies that compared low-fat versus high fat diets predicted 6% increase in triglyceride levels for every 5% decrease in total fat intake [70]. Another meta-analysis of 30 controlled feeding studies in diabetic patients reported greater reductions in triglycerides in response to moderate-fat diets (32.5% to 50% of calories from fat) compared to low-fat diets (18% to 30% of calories from fat) [71]. These inconsistencies may reflect variable uncontrolled fiber, carbohydrate, or protein intake among these studies. On the other hand, several clinical trials reported significant reductions in triglycerides in response to low carbohydrate diets, and a meta-analysis of studies that evaluated low-carbohydrate versus low-fat diets found greater reductions in triglyceride levels in the low-carbohydrate studies [72,73]. Thus, it has been suggested that low-carbohydrate diets exert a more robust triglyceride-lowering effect than low-fat diets [74]. In our current investigation, despite significant improvements in LDL and total cholesterols, triglycerides did not change in response to either diets.

There are several limitations to this study. First, our study is limited in that we had a relatively small sample size (LFWL diet: *n* = 11, LFWM diet: *n* = 10) which imposes a risk of a type II error from low statistical power. However, because we were able to control the actual dietary intake by providing every meal to each participant, the required sample size necessary to detect changes in our primary endpoint (arteriolar FMD) was relatively small. Second, our study represents a short-term intervention which imposed difficulty in identifying long-term consequences of these dietary regimens. Thus, future investigations to evaluate the long-term effects of LFWL and LFWM interventions on endothelial function in health and disease are warranted. Third, risk factors have been eliminated to confine the analysis to the effect of diet on microvascular function which is a strength in design. On the other hand, this design makes it difficult to generalize findings from the current study to obese patients who usually have other risk factors. Finally, one of the major limitations in our study is the unbalanced female to male ratio (2:1). Although this study was not designed to determine gender or racial/ethnic-specific differences in the response to LF diet, future studies are required to determine the influence of such variables on microvascular function.

In conclusion, our findings from this relatively small cohort suggest that LFWL diet was more effective than LFWM diet in improving microvascular health in obese adults despite the improved lipid profile and insulin sensitivity in response to both diets. These findings suggest a major contribution of caloric restriction to microvascular function. The observed microvascular improvements were secondary to an improved local NO production and increased arteriolar sensitivity to NO. Larger, long-term clinical studies are required to investigate the duration and magnitude of dietary effects on endothelial health.

## Figures and Tables

**Table 1:** Subject characteristics.

	LFWL-pre (*n* = 11)	LFWL-post (*n* = 11)	LFWL (Pre-post) *p* ^a^	LFWM-pre (*n* = 10)	LFWM-post (*n* = 10)	LFWM (Pre-post) *p* ^a^	Group Effect (LFWL vs. LFWM) *p* ^b^
Age (year)	36 ± 8			31 ± 6			
Body Weight (kg)	90.7 ± 5.2	87.5 ± 5.1 *	(3.0 ± 0.6) 0.000	101 ± 3.9	101 ± 3.8	(1.1 ± 0.9) 0.270	0.006
BMI (kg/m^2^)	32.6 ± 2.5	29.3 ± 2.9 *	(1.1 ± 0.3) 0.002	35.0 ± 3.9	35.2 ± 3.3	(−0.1 ± 0.6) 0.974	0.039
Body fat %	40.6 ± 1.4	39.5 ± 1.0	(1.9 ± 1.1) 0.105	41.9 ± 1.6	42.4 ± 1.1	(0.2 ± 0.4) 0.653	0.168
Waist Circum. (cm)	93.7 ± 2.7	90 ± 3.1 *	(3.3 ± 0.6) 0.000	107.4 ± 2.4	102.6 ± 3.7	(3.4 ± 2.4) 0.192	0.627
Systolic BP (mm Hg)	112.5 ± 6.8	111.7 ± 9.1	(0.8 ± 3.2) 0.503	120 ± 11.7	118.1 ± 10.3	(2.2 ± 4.4) 0.624	0.292
Diastolic BP (mm Hg)	70.3 ± 9.4	62.2 ± 8.2 *	(8.1 ± 3.1) 0.027	71.4 ± 9.6	70.0 ± 8.1	(1.8 ± 2.6) 0.494	0.055
Heart Rate (beats/min)	68.7 ± 11.3	62.9 ± 7.3	(5.8 ± 3.0) 0.083	63.2 ± 9.8	63.0 ± 10.0	(0.3 ± 2.2) 0.890	0.436
Total Chol (mg/dL)	179.0 ± 12.5	168 ± 11.1 *	(9.5 ± 5.0) 0.041	183 ± 9.5	158 ± 8.9 *	(24.6 ± 6.0) 0.003	0.080
LDL Chol (mg/dL)	104 ± 9.7	100 ± 9.3 *	(4.1 ± 0.7) 0.048	100 ± 7.6	89 ± 7.6 *	(13.2 ± 3.5) 0.004	0.044
HDL Chol (mg/dL)	56 ± 5.7	48 ± 3.9 *	(8.2 ± 2.7) 0.013	59 ± 7.1	49 ± 5.1 *	(10.3 ± 2.4) 0.002	0.479
Triglycerides (mg/dL)	95.2 ± 14.8	96.7 ± 12.1	(6.7 ± 8.5) 0.449	92.0 ± 8.1	92.0 ± 15.2	(7.6 ± 16.5) 0.659	0.870
Glucose (mg/dL)	93.7 ± 14.0	92.5 ± 8.4	(1.3 ± 2.9) 0.663	91.0 ± 12.1	87.8 ± 12.1	(2.4 ± 3.7) 0.534	0.442
Insulin (μIU/mL)	11.6 ± 7.2	6.1 ± 6.5 *	(4.8 ± 1.7) 0.017	13.8 ± 6.7	10.3 ± 7.7	(2.7 ± 1.3) 0.065	0.046
HOMA-IR (AU)	2.6 ± 0.7	1.5 ± 0.4 *	(1.2 ± 0.4) 0.013	3.2 ± 1.1	2.3 ± 0.5 *	(0.7 ± 0.3) 0.036	0.043

*p*^a^ for *p*-value using paired *t*-test (pre-intervention vs. post-intervention); *p* ^b^ for *p*-value using ANCOVA (LFWL vs. LFWM after controlling for the pre-intervention states as baseline covariates); * *p* < 0.05.

**Table 2:** Baseline FID and AchID at the pre- and post-intervention states.

	LFWL-pre (*n* = 11)	LFWL-post (*n* = 11)	LFWL (Pre vs. post) *p* ^a^	LFWM-pre (*n* = 10)	LFWM-post (*n* = 10)	LFWM (Pre vs. post) *p* ^a^	Group Effect (LFWL vs. LFWM) *p* ^b^
Baseline FID Δ10	9.9 ± 1.0	9.1 ± 1.1	0.128	17.1 ± 3.6	22.1 ± 7.6	0.272	0.163
Baseline FID Δ20	24.5 ± 1.7	25.4 ± 1.3	0.469	29.7 ± 4.5	38.9 ± 11.9	0.226	0.088
Baseline FID Δ40	39.7 ± 2.4	41.0 ± 1.3	0.174	45.4 ± 5.9	50.5 ± 10.3	0.332	0.151
Baseline FID Δ60	57.8 ± 2.8	70.2 ± 3.0 *	0.015	65.6 ± 5.7	65.7 ± 12.7	0.497	0.025
Baseline FID Δ100	69.3 ± 1.5	86.2 ± 3.9 *	0.002	83.2 ± 5.2	77.8 ± 12.1	0.334	0.011
Baseline AchID 10^−9^	6.6 ± 1.4	11.4 ± 1.0 *	0.016	8.5 ± 2.1	12.0 ± 2.9	0.167	0.452
Baseline AchID 10^−8^	15.1 ± 2.8	26.6 ± 2.6 *	0.005	16.9 ± 3.3	21.3 ± 4.6	0.221	0.092
Baseline AchID 10^−7^	26.0 ± 3.8	42.5 ± 3.8 *	0.004	30.7 ± 4.9	31.3 ± 6.9	0.473	0.012
Baseline AchID 10^−6^	39.4 ± 4.9	58.6 ± 4.4 *	0.005	48.1 ± 6.9	44.9 ± 10.2	0.397	0.006
Baseline AchID 10^−5^	56.4 ± 5.1	73.8 ± 4.9 *	0.015	64.5 ± 8.5	61.5 ± 13.4	0.422	0.027
Baseline AchID 10^−4^	74.1 ± 6.5	86.4 ± 5.2	0.070	80.7 ± 9.9	80.3 ± 9.4	0.491	0.119

*p*^a^ for *p*-value using paired *t*-test (pre-intervention vs. post-intervention); *p* ^b^ for *p*-value using ANCOVA (LFWL vs. LFWM after controlling for the pre-intervention states as baseline covariates); * *p* < 0.05.

**Table 3:** Absolute changes in the FID after LNAME and indomethacin treatment relative to baseline.

	LFWL-pre (*n* = 11)	LFWL-post (*n* = 11)	LFWL (Pre vs. post) *p* ^a^	LFWM-pre (*n* = 10)	LFWM-post (*n* = 10)	LFWM (Pre vs. post) *p* ^a^	Group Effect (LFWL vs. LFWM) *p* ^b^
LNAME FIDΔ10	−2.9 ± 1.0	−4.3 ± 1.4	0.107	−7.4 ± 1.0	−10.3 ± 2.4	0.261	0.463
LNAME FIDΔ20	−8.4 ± 2.2	−13.7 ± 1.6 *	0.042	−9.6 ± 3.3	−14.3 ± 5.1	0.149	0.301
LNAME FIDΔ40	−9.6 ± 3.2	−22.2 ± 2.9 *	0.021	−16.1 ± 1.1	−19 ± 3.2	0.105	0.051
LNAME FIDΔ60	−14 ± 4.1	−42.5 ± 4.6 *	0.005	−23.3 ± 2.1	−22.6 ± 3.3	0.099	0.002
LNAME FIDΔ100	−14.8 ± 3.0	−46 ± 6.3 *	0.003	−27.4 ± 2.5	−28.1 ± 2.4	0.087	0.000
Indo FIDΔ10	−6.7 ± 3.1	−2.4 ±2.8	0.052	−11 ± 0.7	−7.1 ± 2.0	0.055	0.232
Indo FIDΔ20	−17 ± 7.0	−7.9 ± 1.4 *	0.046	−15.6 ± 3.3	−10.5 ± 2.8	0.084	0.103
Indo FIDΔ40	−26.5 ± 6.9	−13.1 ± 2.9 *	0.040	−20.7 ± 5.6	−9.6 ± 4.1	0.050	0.221
Indo FIDΔ60	−38.6 ± 3.9	−24.5 ± 5.5 *	0.017	−29.5 ± 9.2	−11 ± 4.8 *	0.030	0.069
Indo FIDΔ100	−45.2 ± 7.3	−30.7 ± 4.1 *	0.025	−36.6 ± 11.2	−7.8 ± 5.1 *	0.007	0.012

*p*^a^ for *p*-value using paired *t*-test (pre-intervention vs. post-intervention); *p* ^b^ for *p*-value using ANCOVA (LFWL vs. LFWM after controlling for the pre-intervention states as baseline covariates); * *p* < 0.05; Indo, indomethacin.
